# A Fairy Chemical, Imidazole-4-carboxamide, is Produced on a Novel Purine Metabolic Pathway in Rice

**DOI:** 10.1038/s41598-019-46312-7

**Published:** 2019-07-09

**Authors:** Hirohide Takemura, Jae-Hoon Choi, Nobuo Matsuzaki, Yuki Taniguchi, Jing Wu, Hirofumi Hirai, Reiko Motohashi, Tomohiro Asakawa, Kazutada Ikeuchi, Makoto Inai, Toshiyuki Kan, Hirokazu Kawagishi

**Affiliations:** 1 Graduate School of Science and Technology, Shizuoka University, 836 Ohya, Suruga-ku, Shizuoka, 422-8529 Japan; 2 Graduate School of Integrated Science and Technology, Shizuoka University, 836 Ohya, Suruga-ku, Shizuoka, 422-8529 Japan; 3 Research Institute of Green Science and Technology, Shizuoka University, 836 Ohya, Suruga-ku, Shizuoka, 422-8529 Japan; 4 Institute of Innovative Science and Technology, Tokai University, 4-1-1 Kitakaname, Hiratsuka City, Kanagawa 259-1292 Japan; 5 Department of Chemistry, Faculty of Science, Hokkaido University, Kita 10, Nishi 8, Kita-ku, Sapporo, 060-0810 Japan; 6 School of Pharmaceutical Sciences, University of Shizuoka, 52-1 Yada, Suruga-ku, Shizuoka, 422-8526 Japan

**Keywords:** Biosynthesis, Plant sciences

## Abstract

Rings or arcs of fungus-regulated plant growth occurring on the floor of woodlands and grasslands are commonly called “fairy rings”. Fairy chemicals, 2-azahypoxanthine (AHX), imidazole-4-carboxamide (ICA), and 2-aza-8-oxohypoxanthine (AOH), are plant growth regulators involved in the phenomenon. The endogeny and biosynthetic pathways of AHX and AOH in plants have already been proven, however, those of ICA have remained unclear. We developed a high-sensitivity detection method for FCs including ICA and the endogenous ICA was detected in some plants for the first time. The quantitative analysis of the endogenous level of ICA in rice and Arabidopsis were performed using ^13^C-double labeled ICA. In addition, the incorporation experiment and enzyme assay using the labeled compound into rice and partially purified fraction of rice indicated that ICA is biosynthesized from 5-aminoimidazole-4-carboxamide (AICA), a metabolite on the purine metabolic pathway. The relationship between ICA and AHX was also discussed based on quantitative analysis and gene expression analysis.

## Introduction

“Fairy rings” is a phenomenon in which fruiting bodies of higher fungi occur after turfgrass grows and/or dies in the form of rings^1^. These diameters vary from a few centimeters to more than 15 meters and it is also known as a disease of turfgrass all over the world^2,3^. A term “fairy rings” has its origin in the medieval myths and superstitions associated with their occurrence in the Middle Ages. Since the first scientific report of fairy rings was published in 1675, various studies and reviews have been reported subsequently^1–4^. However, the real identity of the fairies (the causes of growth promotion and suppression) had been a mystery before our study, although there had been an established theory for some time^5,6^. The established theory is that “these fungi usually grow in a radial pattern in the below-ground, decomposing organic matter and releasing nitrogen in the form of ammonia. Ammonia is converted to nitrates, which is readily available to plants, by soil microorganisms, and the abnormal growth of grasses is caused by the accumulation of nitrates. On the other hand, excessive growth of fungi fills the air space in soil and prevents the penetration of water. It causes a drought of the soil, resulting in the death of grasses^2,3^”. However, we thought that the fungi produce specific plant growth regulator(s), therefore, the project for searching for the fairies from a fairy ring-forming fungus was launched. It is now known that more than 50 species of basidiomycetes that form fairy rings and we chose *Lepista sordida* which is one of the major fairy ring-forming fungi in Japan^1,2,7^.

A plant growth-promoting compound, 2-azahypoxanthine (AHX, **1**), and a plant growth-suppressing compound, imidazole-4-carboxamide (ICA, **2**), were isolated from the culture broth of the *L. sordida*, guided by the results in the bioassay using turfgrass^8,9^. The molecular mechanism of the growth regulating activity of **1** has been investigated by using rice that belongs to the same family as turfgrass and showed very similar response to **1**. A few years after the discovery of **1** and **2**, 2-aza-8-oxohypoxanthine (AOH, **3**) was isolated as a metabolite of **1** from **1**-treated rice. This compound also elongated the seedlings of bentgrass and rice like **1**^10^. These three compounds (**1**–**3**) were named fairy chemicals (FCs) after the title of the article in *Nature* which introduced our study^11^. FCs exhibited growth regulatory activity towards all the plants tested regardless of their families (rice, wheat, tobacco, etc.) and conferred tolerance to various and continuous stress (low or high temperature, salt, drought, etc.) on the plants^8–10^. Furthermore, the yields of rice, wheat, and/or other crops increased by treatment with each FCs in greenhouse and/or field experiments, suggesting applications in agriculture^8–10,12,13^.

**Figure 1:**
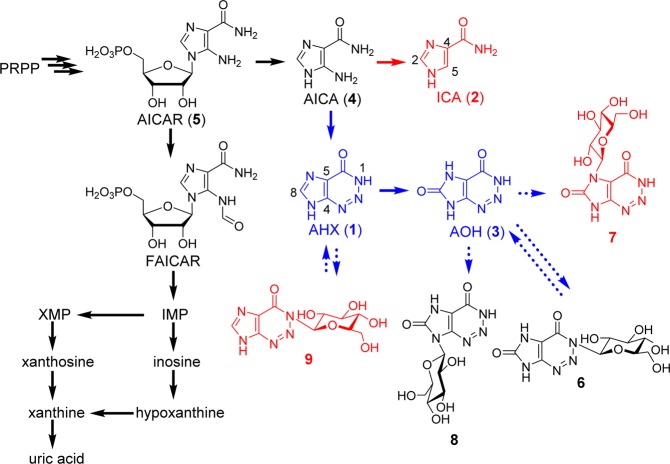
The structures of fairy chemicals and their derivatives, and the novel purine metabolic pathway in rice. The routes indicated by black arrows were adapted from the KEGG (Kyoto Encyclopedia of Genes and Genomes) and those indicated by dash arrows were not clear in the plant. The blue straight arrows and structures show the pathway and metabolites found in the previous study^5,7^. The novel pathway found in this study and the compounds whose endogenous existence have proven in this study are described in red. AHX: 2-azahypoxanthine, AICA: 5-aminoimidazole-4-carboxamide, AICAR: AICA ribonucleotide, AOH: 2-aza-8-oxohypoxanthine, FAICAR: 5-formamidoimidazole-4-carboxamide ribonucleotide, ICA: imidazole-4-carboxamide, IMP: inosine monophosphate, PRPP: phosphoribosyl diphosphate, XMP: xanthosine monophosphate.

FCs are chemically synthesized from a common precursor, 5-aminoimidazole-4-carboxamide (AICA, **4**) (Supplementary Materials Fig. S1)^14–17^. **4** and its ribonucleotide (AICAR, **5**) are common members on the purine metabolic pathway in plants, animals, and microorganisms. **5** is also known as an intermediate of purine bases such as hypoxanthine, xanthine, and uric acid. However, further metabolism of **4** has been unknown (Fig. 1). Therefore, we assumed that plants themselves produce FCs with the same or very similar route of the chemical synthesis. Our data showed the endogenous existence of **1** and **3** in plants and discovered a new route on the purine metabolism in which **1** and **3** are produced from **4** (Fig. 1)^10^. Recently, *N*-glucosides, AOH-1-β-D-glucoside (**6**), AOH-7-β-D-glucoside (**7**), AOH-9-β-D-glucoside (**8**), and AHX-1-β-D-glucoside (**9**) were isolated as the metabolites of **1** and **3** from **1**-treated rice and further metabolisms of **1** and **3** were revealed (Fig. 1)^18^. Contrary to them, molecular mechanism, endogeny, and biosynthetic pathway of **2** in plants had remained unclear. The reason why the research about **2** has less progressed than those of **1** and **3** is that suitable extraction and analytical methods for **2** has not been established.

Here, we established the exhaustive detection method of FCs (**1**–**3**) and their metabolites (**6**–**9**) using LC-MS/MS and proved the universal existence of **2** in plants. The exhaustive quantification of these compounds was also performed. Moreover, the molecular mechanism and the biosynthetic pathway of **2** in rice were discussed based on the oligo DNA microarrays and the incorporation experiments.

## Results and Discussion

### Gene expression profiles of rice treated with 2

It has been reported that **1** and **3** promoted the growth of shoots and roots of rice seedlings and **2** inhibited it^8–10,19^. However, the treatment with not only **1** or **3** but also **2** increased yields of rice and wheat grains^8,12^. The molecular mechanism of growth-regulating activity of **2** was investigated using oligo DNA microarrays of rice containing 42,000 genes (44 K × 4, Agilent Technology). Supplementary Materials Fig. S2 shows the expression profiles of genes that are induced (positive ratios, red coloration) or repressed (negative ratios, blue coloration) by the treatment with each FCs to the control.

The gene expression profile of rice treated with **2** showed largely reverse patterns in comparison to those of **1** and **3** (Supplementary Materials Fig. S2)^9,10^. Treatment with **2** significantly supressed aquaporin (*TIP1;1* and *TIP2;1*) and root-specific genes (root-specific metal transporter and RCc3 protein) (Table 1). Tonoplast intrinsic proteins (TIPs) are the major components of vacuole membranes and the most abundant aquaporins in plants^20^. Especially *Arabidopsis thaliana* aquaporin, AtTIP2;1 has been demonstrated to transport ammonia/ammonium ions, in addition to the water-channel function^21^. It has been reported that root-specific metal transporter and RCc3 play important roles in the regulation of metal homeostasis in *Arabidopsis thaliana* and elongation of root in rice, respectively^22,23^. Hence, the plant growth inhibition by **2**-treatment may be due to the down-regulation of these genes^8,19^. However, the genes of glutathione *S*-transferases (*GST*) were induced by treatment with **2** similar to **1** or **3** (Table 1)^9,10^. It is known that GSTs are involved in the detoxification of xenobiotics, give tolerance to environmental stress to plants, and help protect tissues in various plants. For example, it has been reported that the transgenic Arabidopsis plants expressing rice GST showed better growth under abiotic stress conditions such as heavy metals, low temperature, osmotic pressure, salt, and oxidation^24,25^. Therefore, the fact that the increasing effect of **2** on yields of rice and wheat seeds in the field experiments might be explained by the up-regulation of GSTs.

**Table 1:** Summary of microarray data of rice treated with **1–3**.

Regulated genes	Fold change			
Gene name	ICA (2)		AHX (1)	AOH (3)
	2 μM	10 μM	50 μM	50 μM
Os03g0146100 (tonoplast intrinsic protein1;1, TIP1;1)	−1.79	−3.65	—	2.50
Os02g0658100 (aquaporin TIP2.1)	−2.81	−2.87	2.88	12.1
Os02g0131800 (root-specific metal transporter)	−1.53	−2.37	—	2.31
Os02g0662000 (RCc3 protein)	−2.10	−2.04	2.74	6.61
Os09t0367700 (GST6)	—	5.29	12.0	2.62
Os10t0528300 (Tau GST4)	—	2.46	10.8	4.16

Microarray data of **1** and **3** were cited from ref.^10^.

### Method for extraction and fractionation of 1–3 and 6–9

Both **1** and **2** were chemically synthesized from **4** and it has been reported that **4** converted into **1** in rice^10,14–17^. Hence, we assumed that **2** is also biosynthesized from **4** like **1** in plants. The difficulty in analyzing FCs and their metabolites was due to their low content in plant extracts. In our previous study, repeated fractionations of plant extracts by high-performance liquid chromatography were required for quantification of endogenous **1** and **3**^10^. Therefore, developing a fast and high-sensitivity detection method was desirable for not only **2** but also **1** and **3**.

**Figure 2:**
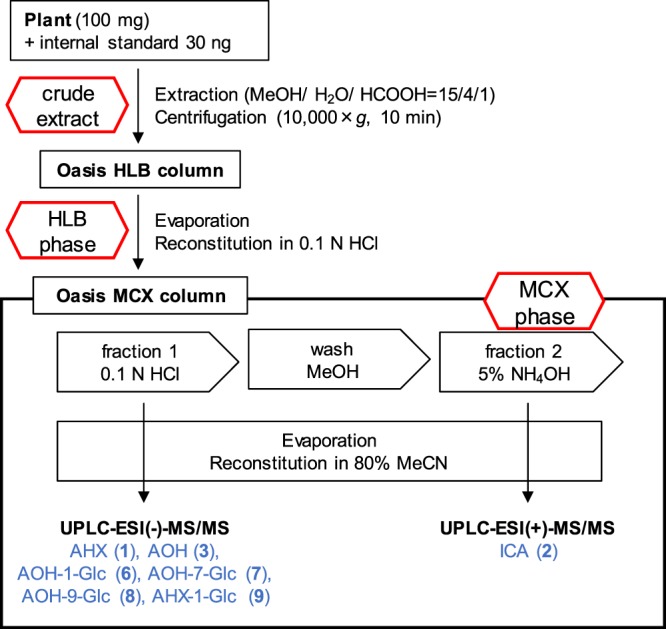
Schematic representation of the extraction and analysis protocol. The compounds were analyzed in LC-MS/MS which shown in blue letters. Each phase was represented in red hexagonal boxes.

Some recently described analytical methods for plant hormones are capable of separating more than ten compounds and these methods are combined solid phase extraction and LC-MS/MS analysis^26,27^. LC-MS/MS can selectively analyze the target compounds based on the mass of precursor ion and product ion, and it is particularly useful for analysis of trace compounds such as plant hormones which are usually at the nM level in plant tissues^26,28^. Hence, we tried to establish efficient detection method of FCs by the reference of the analytical methods of plant hormones and achieved it. The overall procedure for the method summarized in Fig. 2. The extracts were first passed through a solid-phase extraction column (Oasis HLB column) to remove interfering compounds. Since **2** is basic, a strong cation-exchange column (Oasis MCX column) was used as the second column. According to the procedure, **1**, **3**, and **6**–**9** were recovered in the eluate with 0.1 N hydrochloric acid (fraction 1) and **2** was eluted with 5% ammonia solution (fraction 2). The fraction 1 was subjected to LC-MS/MS in the negative ion mode as described previously^10^. On the other hand, the detection sensitivity of **2** was very low in the same analytical conditions for **1** and **3**. Therefore, we newly determined the analytical condition of LC-MS/MS for **2** and the intensity (signal-noise ratio) increased 20-fold in the positive ion mode. The recovery rate of samples after separation on the Oasis MCX column was calculated using authentic standards only and reached on average 95% and 80% for **1**–**3** and **6**–**9**, respectively (Supplementary Materials Table S1). The data showed that **1**–**3** and **6**–**9** were efficiently recovered in this method^27^. Thus, a fast and high-sensitivity detection method for FCs and their derivatives was established.

By using the method, the detection sensitivity of **2** increased about 140-fold from the crude extract (Supplementary Materials Fig. S3). The noteworthy feature of the method is that it required only 100 mg fresh weight (FW) of each plant tissues for detection and enabled fast, high-throughput, and exhaustive analysis of FCs and their derivatives. Moreover, solid phase extraction and separation using an Oasis MCX column have been frequently used as the efficient method for pre-purification of some other plant hormones; acidic plant hormones (indole-3-acetic acid, abscisic acids, and gibberellic acids) were eluted with methanol and alkaline cytokinins were eluted with 0.35 M ammonium hydroxide in 60% methanol^26,29,30^. Therefore, this method has the advantage that analysis of FCs and plant hormone can be performed simultaneously, which will help to clarify the relationship between FCs and plant hormones.

### Quantification of 1–3 in rice and arabidopsis

**Figure 3:**
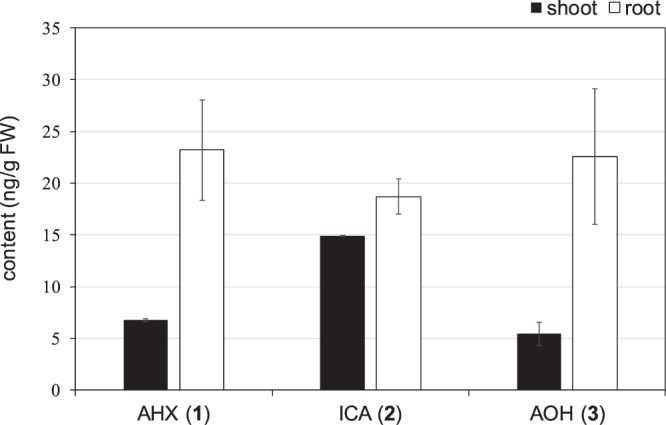
Quantitative analysis of fairy chemicals by LC-MS/MS in rice. The endogenous levels of **1–3** in shoot and root of rice are described as black and white bars, respectively. [4-^13^C,2-^15^N]-**1** and [2,5-^13^C_2_]-**2** were used as internal standards. Results are the mean ± S.D. (n = 3). FW: fresh weight.

To certify the endogeny of **2** in plants, we attempted to analyze various plants and succeeded in the detection of **2** in the plants for the first time (Supplementary Materials Table S2). This result showed that **2** is produced by plants themselves. It has been also reported that **1** and **3** were detected in various plants, indicating that FCs exist in plants universally^10^. Furthermore, the endogenous FCs in rice cultivated under aseptic conditions were quantified. Two stable labeled isotopes [4-^13^C,2-^15^N]-**1** and [2,5-^13^C_2_]-**2**, which were synthesized previously, were utilized for quantification as an internal standard^10,31^. MS detects the molecular ion of a compound and also the molecular ion consisted of naturally occurring stable isotopes whose molecular weight is one or a few larger. Therefore, double isotope labeled compounds are very useful as an internal standard for precise quantification^32^. Furthermore, in this case, labeling carbons of the imidazole ring is very meaningful for elucidation of further metabolism of FCs because their metabolites still having the ring might exist. As a result, the content of FCs in rice was determined as **1** (6.78 ± 0.14 ng/g FW in shoot and 23.2 ± 4.84 ng/g FW in root), **2** (14.9 ± 0.10 ng/g FW in shoot and 18.7 ± 1.69 ng/g FW in root), and **3** (5.45 ± 1.09 ng/g FW in shoot and 22.6 ± 6.57 ng/g FW in root) as shown in Fig. 3. This result indicated that the endogenous level of **2** was similar to those of **1** and **3** and the endogenous levels of FCs were almost the same as those of major plant hormones in rice (gibberellin A_19_; 7.13 ± 0.430 ng/g FW in shoot, indole-3-acetic acid; 24.3 ± 3.81 ng/g FW in shoot), suggesting that FCs regulate plants at the nM level as the plant hormons^26,28^.

Furthermore, **1** (1.25 ± 0.83 ng/g FW in shoot and 0.44 ± 0.12 ng/g FW in root), **2** (5.26 ± 0.62 ng/g FW in shoot and 5.59 ± 1.57 ng/g FW in root), and **3** (731.01 ± 201.37 ng/g FW in shoot and 16.75 ± 6.89 ng/g FW in root) were also detected in Arabidopsis cultivated under aseptic conditions (Supplementary Materials Fig. S4). The endogenous levels of **1** and **2** in Arabidopsis were lower compared to that in rice, whereas that of **3** was much higher in Arabidopsis compared to that in rice. The oxidation from **1** to **3** is catalyzed by xanthine dehydrogenase (XDH), which is known as an enzyme that oxidizes hypoxanthine and xanthine to uric acid in purine metabolism^10,33^. Whereas rice possesses only one gene for *XDH*, Arabidopsis has two genes (*AtXDH1* and *AtXDH2*) and both the genes were more strongly expressed in shoot than root^34^. The differences in the number of *XDH* genes between the two plants species and in expression levels of *XDH* genes between shoot and root in Arabidopsis may have contributed to the high concentration of **3** in the shoot of Arabidopsis.

### Quantification of 6–9 in rice and arabidopsis

Compounds **6**–**9** were isolated from **1**-treated rice, suggesting that plants have a pathway to biosynthesize **6–9** endogenously^18^. Although labeled **6**–**9** have not been chemically synthesized, **1** and **6**–**9** were eluted in the same fraction in the new method (Fig. 1). Then, we tried to determine the quantity of **6**–**9** using [4-^13^C,2-^15^N]-**1** as an internal standard. As a result, **7** (0.17 ± 0.03 ng/g FW in shoot) and **9** (1.42 ± 0.24 ng/g FW in root) were detected in rice (Supplementary Materials Fig. S5 and Table S3). Furthermore, **7** (5.88 ± 1.83 ng/g FW in shoot and 1.27 ± 0.47 ng/g FW in root) was also found in Arabidopsis (Supplementary Materials Table S3). This is the first finding showing that **7** and **9** exist in nature. The content of **7** in Arabidopsis was higher than that in rice and it will be due to a larger amount of **3** in Arabidopsis. The endogenous levels of the glucosides in both rice and Arabidopsis were less than those of FCs themselves. One of the plant hormones, cytokinin, has a purine skeleton like **1** and **3** and it is converted into *N*-glucosides, and the glucosides exhibit little or no activity against plants^18,35^. In terms of their physiological activity, the glucosides are known as transport forms or storage ones^35^. In our previous study, *N*-glucosides **6**–**9** also exhibited no statistically significant activity against rice^18^. Thus, **6**–**9** may also exist as a transport form or a storage form like cytokinin glucosides and the endogenous levels of **6**–**9** may depend on the growth stage.

### The biosynthetic pathway of 2 in rice

As mentioned above, the endogenous existence of **2** in plants was proven. Next step is to certify our hypothetic biosynthetic route from **4** to **2**. Rice seedlings were cultivated in distilled water containing 0.1 mM [2,5-^13^C_2_]-**4** that was synthesized previously, and the extracts of the treated seedlings were analyzed using the new method^31^. The precursor ion (*m/z* 114.0556) and the product ion (*m/z* 97), the same as authentic [2,5-^13^C_2_]-**2**, were detected at the same retention time from shoot and root of the **4**-treated rice (Supplementary Materials Fig. S6), indicating that the biosynthetic route from **4** to **2** indeed exists in rice.

To search for the enzyme(s) that catalyze the conversion of **4** to **2**, the extracts of roots of rice seedlings were ultra-filtrated and the fraction with molecular weight more than 30,000 showed the enzymatic activity for converting [2,5-^13^C_2_]-**4** into [2,5-^13^C_2_]-**2**. This conversion was not observed with the heat-deactivated extracts (Supplementary Materials Fig. S7). In addition, conversion of **4** to **2** did not progress without NaNO_2_. In chemical synthesis of **2** from **4**, **4** is treated with NaNO_2_ and the resulting intermediate, 4-diazo-4*H*-imidazole-5-carboxamide (DICA, **10**), causes deamination by thermolysis (method A) or hydrogenolysis (method B) to give **2** (Supplementary Materials Fig. S1)^14–17,31^. Therefore, the result suggested that **4** was enzymatically converted to **2** in rice and the reaction was very similar to the chemical synthesis.

**Figure 4:**
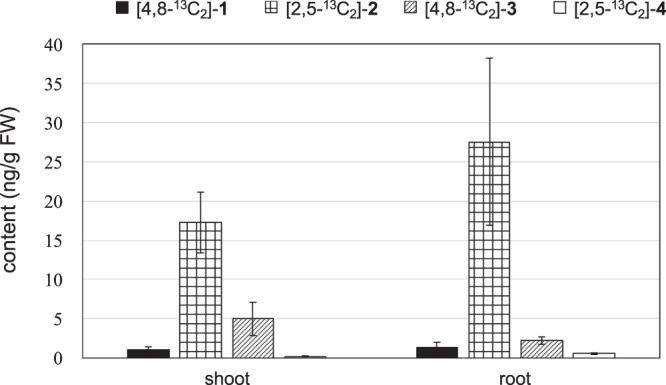
Quantitative analysis of ^13^C_2_ labelled FCs in rice treated with 0.1 mM [2,5-^13^C_2_]-4. The contents of labelled **1–4** in shoot and root of rice treated with 0.1 mM [2,5-^13^C_2_]-**4** is described in left side and right side, respectively. The external standard method was used for quantification. Results are the mean ± S.D. (n = 3). FW: fresh weight.

Finally, the labeled FCs in the rice seedling treated with 0.1 mM [2,5-^13^C_2_]-**4** were quantified (the above experiment) as shown in Fig. 4; [4,8-^13^C_2_]-**1** (1.09 ± 0.36 ng/g FW in shoot and 1.37 ± 0.65 ng/g FW in root), [2,5-^13^C_2_]-**2** (17.3 ± 3.86 ng/g FW in shoot and 27.6 ± 10.6 ng/g FW in root), [4,8-^13^C_2_]-**3** (4.99 ± 2.11 ng/g FW in shoot and 2.44 ± 0.47 ng/g FW in root), and [2,5-^13^C_2_]-**4** (0.23 ± 0.06 ng/g FW in shoot and 0.57 ± 0.10 ng/g FW in root). The concentration of residual [2,5-^13^C_2_]-**4** in the medium after the incubation of rice seedlings was very low (16.8 ± 3.40 nM), while the concentration of the [2,5-^13^C_2_]-**4** in the medium without the seedlings (control) was 95.8 ± 17.8 µM. This result indicated that [2,5-^13^C_2_]-**4** was not decomposed under this culture condition and most of [2,5-^13^C_2_]-**4** was absorbed into rice. However, the content of [2,5-^13^C_2_]-**4** detected in the treated rice was very low and the total amount of labelled FCs was also very small. This result suggested the possibility that [2,5-^13^C_2_]-**4** was converted to other unknown metabolites in rice. In terms of the biosynthesis of FCs, we previously reported that [4-^13^C]-**3** was detected in rice treated with [5-^13^C]-**4** and [4-^13^C]-**1** was not detected in the rice^10^. In this study, the conversion from [2,5-^13^C_2_]-**4** to [4,8-^13^C_2_]-**1** was able to be observed for the first time because the new method is much more sensitive than the previous method. Thus, this result proved our hypothesis that **4** was converted to **3** via **1** in rice. The content of [2,5-^13^C_2_]-**2** was higher than the total contents of [4,8-^13^C_2_]-**1** and [4,8-^13^C_2_]-**3** in both shoot and root, indicating that the metabolism from **4** into **2** is the preferential pathway compared with that from **4** into **1**.

## Conclusion

FCs are plant growth regulators isolated from the fairy-ring fungus as the causative principles of fairy rings. Although the molecular mechanisms, endogeny, and biosynthetic pathways of **1** and **3** in plants have already been proven, those of **2** had not been cleared. It has been reported that **1** and **3**, and **2** exhibited growth-promoting activity and growth-inhibiting activity against rice, respectively^8–10,19^. Here, the gene expression profile of rice treated with **2** showed largely reverse patterns in comparison to those of **1** and **3**, suggesting that the plant growth may be regulated by the endogenous levels of FCs. It was also revealed that the treatment with **2** imparted stress tolerance to plants like **1** and **3**. Furthermore, a detection method for FCs and their derivatives was established. The method enabled fast, high-sensitivity, high-throughput, and exhaustive analysis of FCs and the universal endogeny of the compounds was discovered in plants by using the method. It can contribute to the quantification of FCs in various researches and elucidation of the physiological function of the compounds. The incorporation assay revealed the biosynthetic pathway of **2**, indicating that all FCs are biosynthesized from **4** in rice. The above-mentioned studies and our findings allowed us to conclude that FCs are biosynthesized on the novel purine metabolic pathway in plants and FCs are a candidate of a new family of plant hormones^5,6^.

## Methods

### Materials and chemicals

A rice cultivar (*Oryza sativa* L. cv. Nipponbare) was used for microarrays, qualitative and quantitative analyses of FCs and incorporation assay (Figs 3 and 4, Table 1, Supplementary Materials Figs S2, S3, S5–7 and Tables S2, S3). An Arabidopsis (*Arabidopsis thariana* L. cv. Columbia) was used for qualitative and quantitative analyses of FCs (Supplementary Materials Fig. S4 and Tables S2, S3). A cucumber (*Cucumis sativus* L. cv. Ajisango), lettuce (*Lactuca sativa* L. cv. Cisco), komatsuna (*Brassica rapa* L. cv. Rakuten), tomato (*Solanum lycopersicum* L. cv. House-Momotaro), *Eucalyptus* (*Eucalyptus grandis*) and broccoli (*Brassica oleracea*. L. cv. Green-Beauty) were used for qualitative analysis of **2** (Supplementary Materials Table S2). All solvents used throughout the experiments were obtained from Kanto Chemical Co. (Tokyo, Japan).

### Cultivation of rice

Seeds of rice (*Oryza sativa* L. cv. Nipponbare) were sterilized with 70% (v/v) ethanol for 1 min and 2.5% (v/v) sodium hypochlorite for 10 min. After washing with distilled water for three times, the seeds were transferred to a covered petri dish (90 mm) with 20 mL of distilled water in clean bench and covered from light by aluminum foil. These seeds were incubated for germination at 28 °C. After 3 days, germinated seeds were sown on a net about 50 seeds per a culture pot (*ϕ* 87 × 176.5 mm) with 100 mL of half-strength of nutrient solution A (1 mM NH_4_NO_3_, 0.6 mM Na_2_HPO_4_, 0.3 mM K_2_SO_4_, 0.4 mM MgCl_2_, 0.2 mM CaC1_2_, 45 μM Fe-ethylenediaminetetraacetic acid, 50 μM H_3_BO_3_, 9 μM MnSO_4_, 0.3 μM CuSO_4_, 0.7 μM ZnSO_4_ and 0.1 μM Na_2_MoO_4_). The culture pots were placed in an incubator and maintained in a vertical position under dark/light cycles of 8 h/16 h at 28 °C for a week. Rice seedlings were divided into two parts, shoot and root. All tissues were weighed and stored at −80 °C.

### Cultivation of arabidopsis

Seeds of Arabidopsis (*Arabidopsis thariana* L. cv. Columbia) were sterilized with 70% (v/v) ethanol for 1 min and 1.5% (v/v) sodium hypochlorite for 10 min. After washing with distilled water for three times, the seeds were transferred to a covered petri dish (90 mm) with 20 mL of distilled water in clean bench and covered from light by aluminum foil. These seeds were incubated for germination at 4 °C for 3 days. The germinated seeds were transferred to a new petri dish (90 mm) with 25 mL of half-strength of Murashige-Skoog medium with 10% (w/v) sucrose, 0.5% (w/v) 2-morpholinoethanesulfonic acid monohydrate (MES), 1% (v/v) Gamborg’s vitamin solution and 0.9% (w/v) bacto agar. The final solution was adjusted to pH 5.8. The petri dishes were placed in an incubator and maintained in a vertical position under dark/light cycles of 8 h/16 h at 22 °C for two weeks. Arabidopsis seedlings were divided into two parts, shoot and root. All tissues were weighed and stored at −80 °C.

### Microarray

Immediately after germination, rice seedlings treated with or without **2** were incubated in a test tube for 2 weeks, and half-strength of nutrient solution A was replaced every day. Rice seedlings were divided into two parts, shoot and root. Total RNA was isolated from the seedlings using an RNeasy plant mini kit (Qiagen). cDNA was synthesized to provide 200 ng of total RNA (Agilent Technologies). This cDNA was used as a template to synthesize cRNA. The cRNA was labelled with Cyanine-3 (Cy3) CTP or Cyanine-5 (Cy5) CTP. Cy3-labeled cRNA was mixed with the same amount of Cy5-labeled cRNA and the mixture was used for hybridization. After hybridization for 17 h at 65 °C, the slides were washed and scanned with an Agilent Microarray Scanner. Feature extraction and image analysis software (version A.8.4.1; Agilent Technologies) were used to locate and delineate each spot in the array and to integrate each spot’s intensity, filtering, and normalization. We performed dye swap experiments to estimate the technical and dye labelling error. Hierarchical clustering analyses were performed using Subio Platform ver 1.14 (http://www.subio.jp). Microarray information has been deposited at the Gene Expression Omnibus with the accession number, GSE100899.

### Extraction and fractionation of FCs

The Frozen tissues (about 100 mg FW), and 30 ng of [4-^13^C,2-^15^N]-**1** and [2,5-^13^C_2_]-**2** were added to a 2 mL centrifuge tube filled 1 mL of extraction solvent (methanol: formic acid: water = 15: 1: 4) and crushed tissues using a Micro Smash (4,500 rpm, 3 min) with 3 stainless beads (diameter, 3.2 mm). [4-^13^C,2-^15^N]-**1** and [2,5-^13^C_2_]-**2** were synthesized as reported previously^10,31^. After centrifugation at 10,000 × *g* for 10 min, the supernatant was transferred to a new 2 mL microtube. The pellet was re-extracted with 1 mL of extraction solvent and combined with the first supernatant (approximately 2.0 mL). To remove interfering compounds, the extract was first passed through an Oasis HLB 1 cc/30 mg (Waters) equilibrated with an extraction solvent. After removing the solvent of the extracts under reduced pressure, the residue was reconstituted with 0.5 mL of 2% formic acid. The solution was loaded an Oasis MCX 1 cc/30 mg (Waters) conditioned with methanol and equilibrated with 0.1 N hydrochloric acid, and sequentially eluted with 1 mL of 0.1 N hydrochloric acid (fraction 1), 1 mL of methanol (wash), and then 1 mL of 5% ammonia (fraction 2), and each fraction was evaporated to dry. The fraction 1 that contained **1**, **3**, **6** to **9** and [4-^13^C,2-^15^N]-**1**, and the fraction 2 that contained **2** and [2,5-^13^C_2_]-**2** were dissolved in 120 μL of 0.05% formic acid in 80% acetonitrile and subjected to LC-MS/MS analysis. To determine the recovery rates, ten ng of tested compounds were dissolved in 1 mL of extraction solvent (without plant matrices). The solutions were fractioned as shown in Fig. 2 and each fraction subjected to LC-MS/MS. The results were compared with the tested compounds without fractionations.

### Detection of 2 by LC-MS/MS

All plants were divided into above ground parts and roots. The above ground parts and roots were extracted with ethanol, respectively. After removing the solvent of the extracts under reduced pressure, each sample was dissolved in 80% acetonitrile and subjected to LC-MS/MS analysis.

### UPLC-ESI-Orbitrap MS/MS conditions

A Shimadzu UPLC system (Shimadzu, Japan) coupled to an LTQ Orbitrap mass spectrometry (Thermo Fisher Scientific, Waltham, MA, USA) equipped with an electrospray ionization probe was used. **For 1 and 3;** The analyses were performed according to the method as reported previously^10^. A PC-HILIC column (*ϕ* 2 × 100 mm, 3 μm; Shiseido, Japan) was used in the analysis (injection volume, 10 μL; solvent, 95% acetonitrile with 0.05% formic acid; flow rate; 0.4 mL/min). After each analysis, the column was re-equilibrated for 12 min at the initial conditions prior to the next sample analysis. MS analysis was performed in the negative FTMS mode at a resolution of 30,000 at *m/z* 400 with the following source parameters: sheath gas flow, 50; auxiliary gas flow rate, 10; tube lens, −63 V; capillary voltage, −16 V; ion spray voltage, 3 kV. **For 2;** The LC conditions were the same as those of **1** and **3**. MS analysis was performed in the positive FTMS mode at a resolution of 30,000 at *m/z* 400 with the following source parameters: sheath gas flow, 50; auxiliary gas flow rate, 10; tube lens, −14.7 V; capillary voltage, 1 V; ion spray voltage, 3 kV. **For 6 to 9;** A InertSutain AQ-C18 column (*ϕ* 2 × 100 mm, 3 μm; GL science, Japan) was used in the analysis (injection volume, 10 μL; solvent, 2% methanol with 0.05% formic acid; flow rate; 0.2 mL/min). After each analysis, the column was re-equilibrated for 12 min at the initial conditions prior to the next sample analysis. MS analysis was performed in the negative FTMS mode at a resolution of 30,000 at *m/z* 400 with the following source parameters: sheath gas flow, 50; auxiliary gas flow rate, 10; tube lens, −32 V; capillary voltage, −4 V; ion spray voltage, 3 kV. MS spectra were detected by Orbitrap Fourier transform mass spectrometer (Orbitrap FT-MS) and MS/MS spectra were detected by linear ion trap quadrupole mass spectrometer (LTQ-MS). Compounds were identified by exact mass and characteristic transitions (precursor ion to daughter ion).

### Incorporation assay

For the aseptic culture of rice, all the procedures were performed in sterile environments. Sterilized seeds of rice were germinated in the dark at 30 °C for 3 days. Then, transferred each four seedlings to test tubes (30 × 200 mm) containing distilled water with 0.1 mM [2,5-^13^C_2_]-**4** for 7 days. [2,5-^13^C_2_]-**4** was synthesized previously^31^. The seedlings were grown under light at 30 °C. Culture media were collected after seedlings were incubated for 7 days. Negative controls were incubated in the same way without rice seedlings. The filtered media was dryed in a centrifugal evaporator and rice seedlings were divided into two parts, shoot and root. All samples were prepared for LC-MS/MS analysis using the sample preparation procedure.

### Enzyme preparation and assay

Three grams of frozen roots of rice was extracted with buffer A (100 mM MES-NaOH, pH 6.0, 0.1% (w/v) CHAPS, 10% glycerol, 1 mM PMSF, 1 mM DTT) in a test tube containing a stainless-steel bead and milled using a beads beater. The extract was centrifuged at 10,000 × *g* for 30 min. After centrifugation, the supernatant was placed in dialysis with tubing with an MWCO value of 14,000 in buffer B (20 mM MES-NaOH, pH 6.0). The resulting supernatant with molecular weight over 30 kDa obtained by ultrafiltration (Amicon Ultra-15, 30,000 MWCO). Assays were performed to confirm the enzymatic activity converting **4** into **2**. 2 μL of 2 mM [2,5-^13^C_2_]-**4** and 1 μL of 10 mM NaNO_2_ dissolved was added to 20 μL of the enzyme in buffer B in a 1.5 mL tube, and the reaction mixture was incubated at 30 °C for 3 h. The reaction was stopped by boiling and the mixture was centrifuged at 15,000 × *g* for 20 min. The converting activity of the crude enzyme was analyzed by LC-MS/MS analysis in the positive mode. An ADME column (*ϕ* 2 × 100 mm, 3 μm; Shiseido, Japan) was used in the analysis (injection volume, 10 μL; solvent, 98% ammonium formate; flow rate; 0.2 mL/min).

## Supplementary information

Supplementary materials
