# Supplementary material for: A Fairy Chemical, Imidazole-4-carboxamide, is Produced on a Novel Purine Metabolic Pathway in Rice
Source: Sci Rep. 2019 Jul 9;9:9899. doi: 10.1038/s41598-019-46312-7 (PMC6616479; doi:10.1038/s41598-019-46312-7)
Supplement: Supplementary file 1 — Supplementary materials [file 41598_2019_46312_MOESM1_ESM.docx]

**Supplementary materials**

**A Fairy Chemical, Imidazole-4-carboxamide, is Produced on a Novel Purine Metabolic Pathway in Rice**

**Hirohide Takemura^1^, Jae-Hoon Choi^2,3^, Nobuo Matsuzaki^1^,** **Yuki Taniguchi^2^, Jing Wu^3^, Hirofumi Hirai^2,3^, Reiko Motohashi^2,3^, Tomohiro Asakawa^4^, Kazutada Ikeuchi^5^, Makoto Inai^6^, Toshiyuki Kan^6^, and Hirokazu Kawagishi^1,2,3,^***

^1^Graduate School of Science and Technology, ^2^Graduate School of Integrated Science and Technology, and ^3^Research Institute of Green Science and Technology, Shizuoka University, 836 Ohya, Suruga-ku, Shizuoka 422-8529, Japan

^4^Institute of Innovative Science and Technology, Tokai University, 4-1-1 Kitakaname, Hiratsuka City, Kanagawa 259-1292, Japan

^5^Department of Chemistry, Faculty of Science, Hokkaido University, Kita 10, Nishi 8, Kita-ku, Sapporo 060-0810, Japan

^6^School of Pharmaceutical Sciences, University of Shizuoka, 52-1 Yada, Suruga-ku, Shizuoka 422-8526, Japan

*kawagishi.hirokazu@shizuoka.ac.jp

**Supplementary Fig. S1. Chemical synthesis of FCs.** Methods A to D were described in references 16, 31, 15 and 17, respectively. Conversion of **1** to **3** was carried out with xanthine oxidase (XOD) from buttermilk in 10 mM phosphate-buffered saline (PBS), pH 7.4.^17^

**Supplementary Fig. S2.** **Gene expression profiles of rice treated with 1, 3 and 2.** Hierarchical clustering analysis was performed using Subio Platform ver 1.14 (Subio). Sample tree is at the top and gene tree is at left. The color bar at right shows levels of expression: red indicates up-regulated genes and blue indicates down-regulated ones to the control (log_2_ ratio). The horizontal axis shows samples (treating substances, concentration of each substance, root/shoot and dye-swap to normalize for dye effect). The gene expression profile data of **1** and **3** were cited from reference 10.

**Supplementary Fig. S3. The relative detection sensitivity of 2 in each phase.** [2,5-^13^C_2_]-**2** in the eluate were analyzed by LC-MS/MS. The values show the relative detection sensitivity compared with crude extracts.

**Supplementary Fig. S4.** **Quantitative analysis of fairy chemicals by LC-MS/MS** **in Arabidopsis.** The endogenous levels of **1**–**3** in shoot and root of Arabidopsis are described as black and white bars, respectively. [4-^13^C,2-^15^N]-**1** and [2,5-^13^C_2_]-**2** were used as internal standards. Results are the mean ± S.D. (n = 3). FW: fresh weight.

**Supplementary Fig. S5. Detection of FCs metabolites by FCs detection method.** LC-MS (bottom) and LC-MS/MS (top) chromatogram of the endogenous FCs metabolites which were newly detected in this study. MS spectra were detected by Orbitrap Fourier transform mass spectrometer (Orbitrap FT-MS) and MS/MS spectra were detected by linear ion trap quadrupole mass spectrometer (LTQ-MS). Compounds were identified by the exact mass and characteristic transitions (precursor ion to daughter ion). MS chromatograms and spectra of; (a) authentic **6**, (b) authentic **7**, (c) authentic **8**, (d) authentic **9**, (e) **7** from rice shoot, (f) **9** from rice root. Compound **6** and **8** were not detected from any part of rice.

**Supplementary Fig. S6.** **Detection of [2,5-^13^C_2_]-2 in rice at day 7 after treatment with 0.1 mM [2,5-^13^C_2_]-4.** LC-MS (bottom) and LC-MS/MS (top) chromatograms and MS spectra of a fraction 2 from shoot (a), root (b), and authentic sample (c). MS spectra were detected by Orbitrap Fourier transform mass spectrometer (Orbitrap FT-MS) and MS/MS spectra were detected by linear ion trap quadrupole mass spectrometer (LTQ-MS). Compounds were identified by exact mass and characteristic transitions (precursor ion to daughter ion).

**Supplementary Fig. S7. Detection of [2,5-^13^C_2_]-2 in reaction mixture of [2,5-^13^C_2_]-4 and crude enzyme from rice.** LC-MS/MS chromatograms of reaction mixture of [2,5-^13^C_2_]-**4** and crude enzyme were obtained by selected reaction monitoring (SRM) for [2,5-^13^C_2_]-**2**.

**Table S1. The recovery rate on the method.**

| compound | recovery rate (%) |
| --- | --- |
| **1** | 95 ± 2 |
| **2** | 94 ± 1 |
| **3** | 95 ± 1 |
| **6** | 82 ± 8 |
| **7** | 80 ± 9 |
| **8** | 84 ± 5 |
| **9** | 84 ± 4 |

The recovery rate of **1**-**3** and **6**-**9** from standard solutions without plant matrices following the procedure. An absolute amount of 10 ng was used.

**Table S2. Detection of 2 in plants by LC-MS/MS.**

| Plant | culture condition | tissue | ICA (**2**) |
| --- | --- | --- | --- |
| rice | aseptic | shoot | d |
|  |  | root | d |
|  | non-aseptic | chaff  endosperm | d  d |
| Arabidopsis | aseptic | shoot | d |
|  |  | root | d |
| cucumber | aseptic | shoot | d |
|  |  | root | d |
| lettuce | aseptic | shoot | d |
|  |  | root | d |
| komatsuna (*Brassica rapa*) | aseptic | shoot | d |
|  |  | root | d |
| tomato | aseptic | shoot | d |
|  |  | root | d |
| *Eucalyptus* | non-aseptic | shoot | d |
|  |  | root | d |
| broccoli | non-aseptic | shoot | d |
|  |  | root | d |

Compound **2** was identified on LC-MS/MS by comparing the retention time, precursor ion and mass transitions with those of standards. d: detected.

**Table S3. Quantification of 6 to 9 in rice and Arabidopsis by LC-MS/MS.**

| compound | shoot of rice | root of rice | shoot of Arabidopsis | root of Arabidopsis |
| --- | --- | --- | --- | --- |
| **6** | nd | nd | nd | nd |
| **7** | 0.17 ± 0.03 ng/g FW | nd | 5.88 ± 1.83 ng/g FW | 1.27 ± 0.47 ng/g FW |
| **8** | nd | nd | nd | nd |
| **9** | nd | 1.42 ± 0.24 ng/g FW | nd | nd |

All the compounds were identified on LC-MS/MS by comparing the retention time, precursor ion and mass transitions with those of standards. [4-^13^C,2-^15^N]-**1** was used as the internal standard. FW: fresh weight, nd: not detected.
